# Investigation of Altered Spontaneous Brain Activities in Patients With Moyamoya Disease Using Percent Amplitude of Fluctuation Method: A Resting-State Functional MRI Study

**DOI:** 10.3389/fneur.2021.801029

**Published:** 2021-12-24

**Authors:** Chu-Qi Li, Qian-Min Ge, Hui-Ye Shu, Xu-Lin Liao, Yi-Cong Pan, Jie-Li Wu, Ting Su, Li-Juan Zhang, Rong-Bin Liang, Yi Shao, Er-Ming Zeng

**Affiliations:** ^1^Department of Neurosurgery and Ophthalmology, The First Affiliated Hospital of Nanchang University, Nanchang, China; ^2^The First Clinical Medical College, Nanchang University, Nanchang, China; ^3^Department of Ophthalmology and Visual Sciences, The Chinese University of Hong Kong, Shatin, Hong Kong SAR, China; ^4^Fujian Provincial Key Laboratory of Ophthalmology and Visual Science, Department of Ophthalmology, Eye Institute of Xiamen University, Xiang'an Hospital of Xiamen University, Xiamen University School of Medicine, Xiamen, China; ^5^Department of Ophthalmology, Massachusetts Eye and Ear, Harvard Medical School, Boston, MA, United States

**Keywords:** percent amplitude of fluctuation (PerAF), resting-state functional magnetic resonance imaging (rs-fMRI), retinal nerve fiber layer thickness (RNFLT), anxiety, depression, moyamoya disease (MMD)

## Abstract

**Background:** Moyamoya disease (MMD) is a chronic progressive cerebrovascular abnormality characterized by chronic occlusion of large intracranial vessels with smoky vascular development at the base of the skull. In patients with MMD, abnormal spontaneous brain activity would be expected.

**Purpose:** To assess the brain activity changes in patients with MMD by resting-state functional MRI (rs-fMRI), using the percent amplitude of fluctuation (PerAF) analysis method.

**Materials and Methods:** A total of 17 patients with MMD (3 males and 14 females) and 17 healthy control (HC) subjects with matched gender and age were recruited for this study. We used rs-fMRI to scan all the patients with MMD. Spontaneous neural activity was evaluated using the PerAF approach. The receiver operating characteristic (ROC) curve analysis was used to assess the ability of the PerAF to distinguish patients with MMD from HCs. The Hospital Anxiety and Depression Scale (HADS) tests were performed to assess the emotional status of patients with MMD and retinal nerve fiber layer thickness (RNFLT) was measured using high-resolution optical coherence tomography (hr-OCT). The relationship between the HADS scores, RNFLT values, and the PerAF signals was assessed using the Pearson's correlation analysis.

**Results:** Compared with HCs, the PerAF signals in patients with MMD were decreased in the Frontal_Sup_Medial_R and Precentral_L, whereas those in the Caudate_L were increased. The areas under the ROC curves indicated that signals in these brain regions could distinguish between patients with MMD and HCs. The PerAF value of Frontal_Sup_Medial_R was positively correlated with the left and right eye RNFLT values and negatively correlated with the HADS scores.

**Conclusion:** In patients with MMD, reduced PerAF signals in the Frontal_Sup_Medial_R, Precentral_L, and Caudate_L may be associated with psychiatric diseases including anxiety and depression and decreased RNFLT may be associated with ophthalmic complications due to the compression of terminal branches of the internal carotid artery in the retinal fiber layer. The PerAF can be used as an effective indicator of ocular complications of MMD and to study the neural mechanism underpinning emotional complications in patients with MMD.

## Introduction

**Figure 1:**
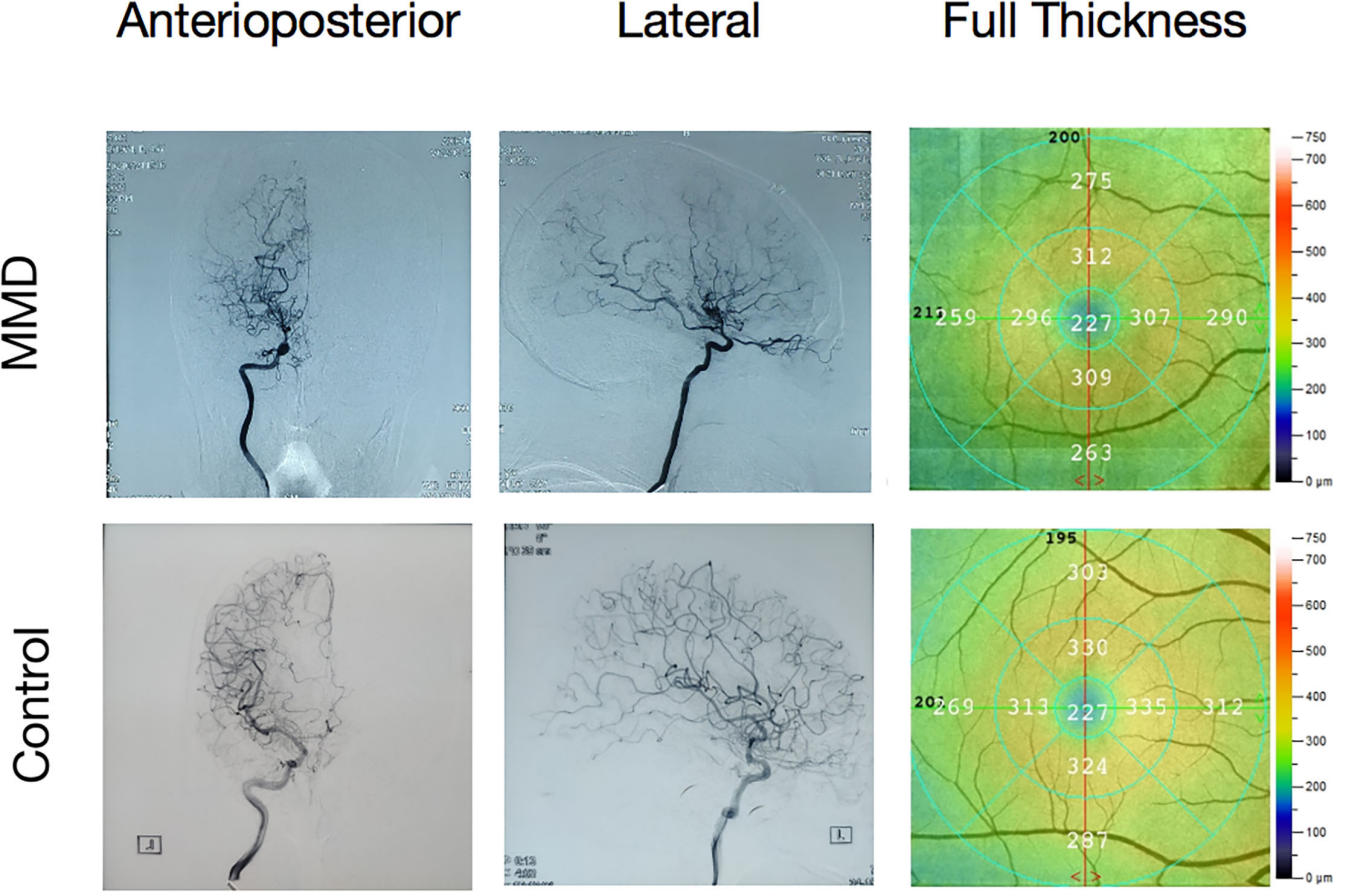
A moyamoya disease (MMD) angiography illustration and a fundus camera illustration. Internal carotid in the digital subtraction angiography (DSA). Undeveloped anterior and middle cerebral arteries and multiple anomalous vascular networks at the base of the skull.

Moyamoya disease (MMD) is a non-atherosclerotic cerebrovascular structural abnormality, which results from progressive stenosis of the intracranial internal carotid arteries and their proximal branches, with insufficient blood supply to the supraclinoid anterior circulation. Due to compensation for cerebral ischemia (1), patients with MMD will have abundant growth of collateral vessels, which contribute to most of the clinical symptoms of MMD. The main complications of MMD include transient ischemic attack (TIA), hemiparesis, hemorrhagic stroke, seizure, epilepsy, and other symptoms caused by repeated cerebral ischemia and stroke such as memory loss, headache, and progressive loss of cognitive ability (2). According to studies conducted between 1997 and 2011, MMD has a 10-fold higher prevalence in Asian populations than in other ethnic groups. For example, prevalence rates of 1/280,000 have been reported in China and 1/89,000 in Japan, but 1/1,100,000 in the US and 1/2,130,000 in Denmark and prevalence is increasing in both the East Asia and the US (2–5). As the most common pediatric cerebrovascular disease in East Asian regions, MMD is mostly diagnosed in children between 10 and 14 years old or in adults after 40 years old, with females at higher risk (6) (Figure 1).

Digital subtraction angiography (DSA) is widely used in clinical practice and allows blood vessels to be visualized through bone and soft tissues. Doctors can obtain the image as the difference between images before and after administration of the contrast medium. Since its first use in the 1970's, DSA has been widely used in the diagnosis of MMD and is considered to be the gold standard. Some studies indicate that the nephrotoxic contrast agent may cause kidney damage, but X-ray is also harmful for patients, so DSA cannot provide dynamic information and references to clinicians in the diagnosis and treatment of MMD. Functional MRI (fMRI) is a widely used imaging technique, which can detect structural changes in brain regions of interest (ROI) at rest. It is non-invasive and can produce three-dimensional (3D) images without harmful X-rays or nephrotoxic contrast agents (7) and there are no reports of ionizing radiation injury in patients with MMD who have undergone MRI scanning. MRI scanning can also provide images with high tissue structure resolution and its multiple sequencing can provide information to help localize the disease lesion and determine its size (8). The diagnostic signs of MMD in an MRI scan include disappearance of the occlusive vascularity effect and increased vascularity in collateral circulation (9, 10) fMRI has been applied in investigation of the neural mechanisms of neurological diseases. The percent amplitude of fluctuation (PerAF) is a new method of fMRI analysis. Instead of measuring the blood oxygenation level-dependent (BOLD) signal at voxel level, the PerAF reflects the percent signal change by measuring the percentage of BOLD fluctuations relative to the mean BOLD signal intensity for each time point and averaging across the whole time series that directly reflects the resting-state BOLD signal fluctuations (11). It is not influenced by the raw signals due to it do not contain arbitrary units, so the PerAF values are less affected by signal strength errors, more accurate and more suitable for ensuing statistical analysis than other MRI analysis methods such as amplitude of low frequency fluctuation, regional homogeneity, and degree centrality (12–14). In addition, the PerAF can be used for group-level statistical analysis and is unaffected by the confounding mixture of voxel-specific fluctuation amplitude in the amplitude of low-frequency fluctuation method (11). For these reasons, the PerAF can measure voxel-level brain activity changes with higher accuracy and efficiency and is a promising analysis method of voxel-level spontaneous BOLD activity. So far, the PerAF has not been used to explore the pathogenesis and clinical complications of cerebrovascular diseases.

Currently, DSA is the main diagnostic method of MMD and study on brain anomalies in MMD is lacking. We speculate that using the PerAF method to study changes of brain activity in MMD will provide information for diagnosis and the underlying neurological basis including biological indicators. In this study, we used the PerAF analysis to assess spontaneous brain activity and mean retinal nerve fiber layer thickness (RNFLT) in sufferers with MMD. We examined the association between the PerAF signals and clinical characteristics, which may help to reveal the underlying neural mechanisms of MMD and help clinicians to predict the disease and protect their patients from potential complications.

## Materials and Methods

### Subjects

A total of 17 sufferers with MMD (3 males and 14 females) were enrolled in this study. The inclusion criteria were: (1) Patients with typical bilateral MMD diagnosed using DSA; (2) No contraindications to head scan of MRI such as implanted metal devices; and (3) Age between 18 and 60 years. The exclusion criteria were: (1) Patients with atypical MMD or moyamoya syndrome; (2) History of craniocerebral surgery; (3) Severe medical disease and visual and hearing impairment; (4) History of stroke; (5) Addiction (for example, to drugs or alcohol); and (6) Contraindications to MRI examination.

This study also enrolled 17 healthy control (HC) subjects (4 males and 13 females) matched for age, gender, and education level. The inclusion criteria were: (1) No MMD diagnosis; (2) No other defects in brain function (such as cerebrovascular diseases and tumors); (3) No serious organic diseases such as heart disease or hypertension; (4) No history of neurologic or psychiatric disorders (anxiety disorders or delusions); and (5) No contraindication to MRI examination. The RNFLT values were measured at baseline, using high-resolution optical coherence tomography (hs-OCT), following a previously described protocol (15).

The Ethics Committee of the First Affiliated Hospital of Nanchang University approved this study. In this study, all the procedures followed the principles of the Declaration of Helsinki and Medical Ethics guidelines. Every subject voluntarily underwent the study procedures and was informed about the aim of this study as well as the potential risks. Each subject signed an informed consent form before recruitment.

### Digital Subtraction Angiography

Digital subtraction angiography of the brain was performed. A digital subtraction vascular machine was used. The patient was instructed to adopt the supine position, the towel was disinfected, femoral artery puncture was performed, and a 5F sheath was inserted 2 cm below the inguinal ligament of the lower extremity of patient. A Pigtail catheter was inserted into the aortic arch and 30 ml contrast agent was injected into the aortic arch at a constant rate. The Pigtail catheter was replaced and a 5F vertebral artery angiography tube was inserted into the common carotid artery, internal carotid artery, subclavian artery, and vertebral artery along with the arterial sheath and DSA was performed in the anteroposterior, lateral, oblique, and Torontonian positions.

### Resting-State FMRI (Rs-FMRI) Protocol

Magnetic resonance imaging scans in this study were implemented on a Trio 3-Tesla MRI scanner using the total imaging matrix method (Siemens, Berlin, UK). All the subjects were instructed to breathe steadily without moving their heads during the scan. To obtain T1-weighted images, all the subjects were scanned using parameters reported in previous studies (16).

### Resting-State FMRI Data Analysis

The functional data were prefiltered to remove incomplete data with MRIcro program (https://crnl.readthedocs.io/). We deleted the first 10 volumes from each data of subject to balance the signal. Then, we use Data Processing Assistant for rs-fMRI version 4.0 (http://rfmri.org/DPARSF) and Statistical Parametric Mapping version 8 (http://www.fil.ion.ucl.ac.uk/spm) for head movement correction and spatial normalization; then, we exported the data to a Digital Imaging Communication System. The images were smoothened with 6 × 6 × 6 mm^3^ voxel size and full width of 6 mm. The scan data of the participants with head movement over 3 mm were deleted and head movement correction was applied to other valid data (17). The automated anatomical labeling (AAL) was used as a template in the study. Linear regression would filtered out the false variable values (18) and we use echo plane image template to standardize the fMRI scans, with the guidance of the Montreal Neurological Institute standards.

The PerAF method used in this study differs from that of previous study. The average BOLD signal values were calculated on the basis of measurements and the percentage of BOLD fluctuation was measured at different time points in relation to the average BOLD value, which was then averaged over the whole time series to acquire the PerAF. The PerAF value for each voxel was calculated using the equations below.


(1) $$\begin{array}{l} \;PerAF=\frac{1}{n}\overset{n}{\underset{n=1}{\sum }}|\frac{x_{i}-\mu }{\mu }|\times 100\% \end{array}$$

(2) $$\begin{array}{l} \;\mu =\frac{1}{n}\overset{n}{\underset{i=1}{\sum }}x_{i} \end{array}$$

In these equations, *x*_*i*_ is the signal strength at the *i*_*th*_ time point, *n* is the number of all the time points, and μ is the average value across the time series.

### Brain–Behavior Correlation Analysis

Brain regions were classified into ROIs based on the PerAF findings, with the resting state fMRI data analysis toolkit (REST) program (https://sourceforge.net/projects/resting-fmri/). The average PerAF value of each ROI was extracted by averaging the PerAF values on all the voxels. Lastly, the relationship between the average PerAF signal of each brain region of MMDs and corresponding functional abnormalities was discussed through correlation analysis.

All the participants completed the Hospital Anxiety and Depression Scale (HADS) questionnaire and the result was evaluated by the Pearson's correlation analysis (*p* < 0.05). The SPSS software version 20.0 (IBM Corporation, Armonk, New York, USA) were used to perform linear correlation.

### Statistical Analysis

After controlling for age and sex, the SPSS software (IBM Corporation, Armonk, New York, USA) was used to perform independent samples *t*-tests comparing the demographic data, RNFLT, and visual acuity (VA) between patients with MMD and HCs. *p* < 0.05 was considered as statistically significant. The 2-sample *t*-test was used to evaluate the differences in the PerAF values between the two groups using the REST program. The Gaussian random field theory was applied to determine the voxel-wide threshold in multiple comparisons as *p* < 0.005. The AlphaSim correction was also applied to clusters over 103 voxels. The receiver operating characteristic (ROC) curve analysis was performed to compare the average PerAF between the two groups using the area under the ROC curve (AUC). The Pearson's correlation analysis was utilized to evaluate the associations between the PerAF values in clusters and the HADS scores as well as RNFLT.

## Results

### Demographics and Clinical Indicators

No significant differences in age (unpaired *t*-test, *p* = 0.90), sex (chi-squared test, *p* > 0.99), or body weight (unpaired *t*-test, *p* = 0.917) were found between the MMD and HC groups. VA-left (*p* = 0.768) and VA-right (*p* > 0.99) also showed no significant differences between the groups. However, significant between-group differences were found in RNFL thickness of the right (*p* < 0.001) and left eye (*p* < 0.001) (Table 1).

**Table 1:** Basic information of participants in the study.

**Condition**	**MMD**	**HCs**	**t**	P-value^*^
Male/female	3/14	3/14	N/A	>0.99
Age (years)	50.11 ± 5.58	49.88 ± 5.29	0.126	0.90
Weight (kg)	61.22 ± 5.12	60.12 ± 9.15	0.196	0.917
Handedness	17R	17R	N/A	>0.99
Duration of MMD (Mons)	3.12 ± 0.24	N/A	N/A	N/A
Best-corrected VA-left eye	0.029 ± 0.014	0.023 ± 0.013	0.298	0.768
Best-corrected VA-right eye	0.064 ± 0.019	0.064 ± 0.019	<0.001	>0.99
IOP-Left	15.05 ± 0.40	14.86 ± 0.34	0.363	0.719
IOP-Right	15.94 ± 0.39	15.26 ± 0.42	1.165	0.253
RNFLT-right (μm)	117.06 ± 17.35	138.76 ± 12.07	4.235	<0.001
RNFLT-left (μm)	115.76 ± 19.22	136.41 ± 11.63	3.789	<0.001^#^
Higher education/HSEB	8/9	8/9	N/A	N/A

*MM, independent t-tests comparing two groups (p <0.05); HCs, healthy controls; N/A, not applicable*.

*Significant at*

* *P <0.05*;

# *P <0.001*.

### Percent Amplitude of Fluctuation in MMD

**Figure 2:**
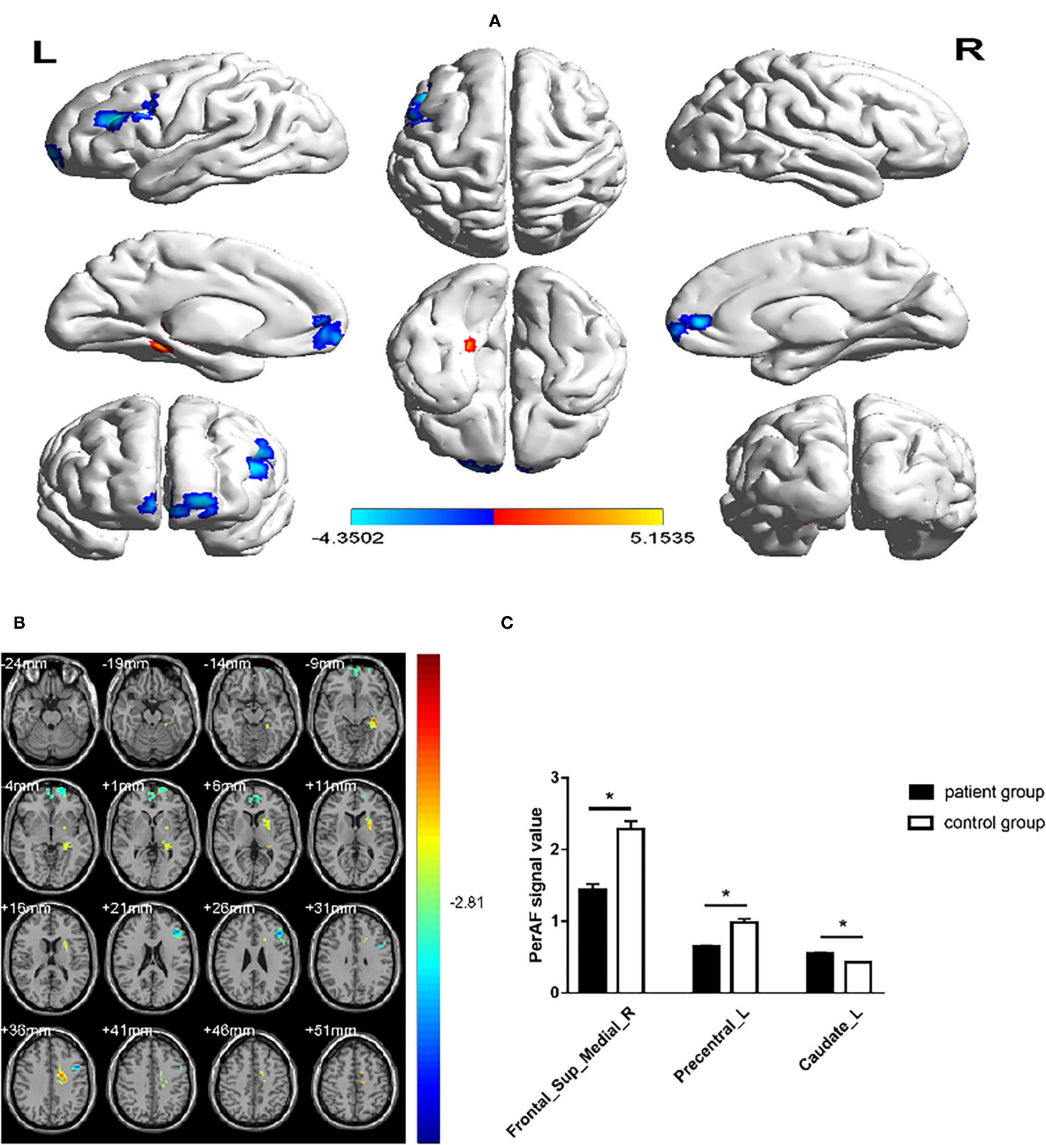
Comparison of the PerAF signal in patients with MMD and HCs. **(A,B)** Green and orange represent the signal strength. The PerAF value in Frontal_Sup_Medial_R and Precentral_L was decreased, while the PerAF value was increased in Caudate_L (the AlphaSim correction was performed at cluster size > 103 voxels, *p* < 0.005). **(C)** The mean PerAF values in the 2 groups. MMD, moyamoya disease; PerAF, percent amplitude of fluctuation; HC, healthy control. *P < 0.05.

The PerAF values in Frontal_Sup_Medial_R and Precentral_L regions were significantly lower in MMD than in HC, while values in the Caudate_L were higher in MMD than in HC (Figure 2, Table 2).

**Table 2:** Brain areas with significantly different AF values between patients with MMD and HCs.

**Brain areas**	**MNI coordinates**			**BA**	**Number of voxels**	**T value**
	**X**	**Y**	**Z**			
**Patient < HC**						
Frontal_Sup_Medial_R	6	51	3	24	159	−3.8795
Precentral_L	−48	9	36	1	135	−4.3502
**Patient > HC**						
Caudate_L	−18	15	9	0	104	3.8544

*The statistical threshold was set at a voxel level with p <0.005 for multiple comparisons using Gaussian random field theory (the AlphaSim corrected at cluster > 103 voxels, p <0.05). PerAF, percent amplitude of fluctuation; HC, healthy control; MNI, Montreal Neurological Institute; MMD, moyamoya disease*.

### Receiver Operating Characteristic Curve

**Figure 3:**
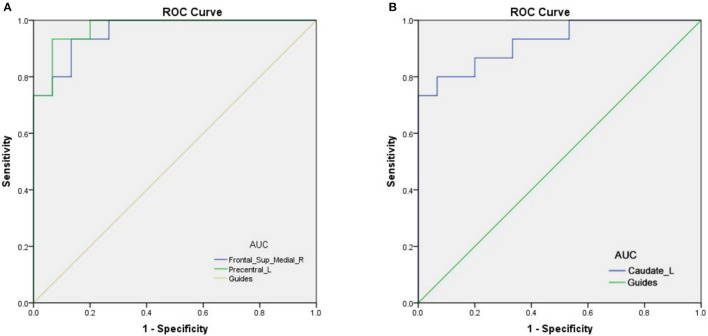
The ROC curve analysis of the mean PerAF values of different brain regions. **(A)** The AUCs for Frontal_Sup_Medial_R and Precentral_L were 0.960 (*p* < 0.0001; 95% CI: 0.900–1.000) and 0.973 (*p* < 0.0001; 95% CI: 0.926–1.000), respectively **(B)** The AUC was 0.924 (*p* < 0.0001; 95% CI: 0.831–1.000) for Caudate_L. ROC, receiver operating characteristic; AUC, area under the ROC curve; MMD, moyamoya disease; PerAF, percent amplitude of fluctuation.

The AUCs for Frontal_Sup_Medial_R and Precentral_L were 0.960 (*p* < 0.0001; 95% CI: 0.900–1.000) and 0.973 (*p* < 0.0001; 95% CI: 0.926–1.000), respectively, and the AUCs for Caudate_L was 0.924 (*p* < 0.0001; 95% CI: 0.831–1.000) (Figure 3).

### Correlation Analysis

**Figure 4:**
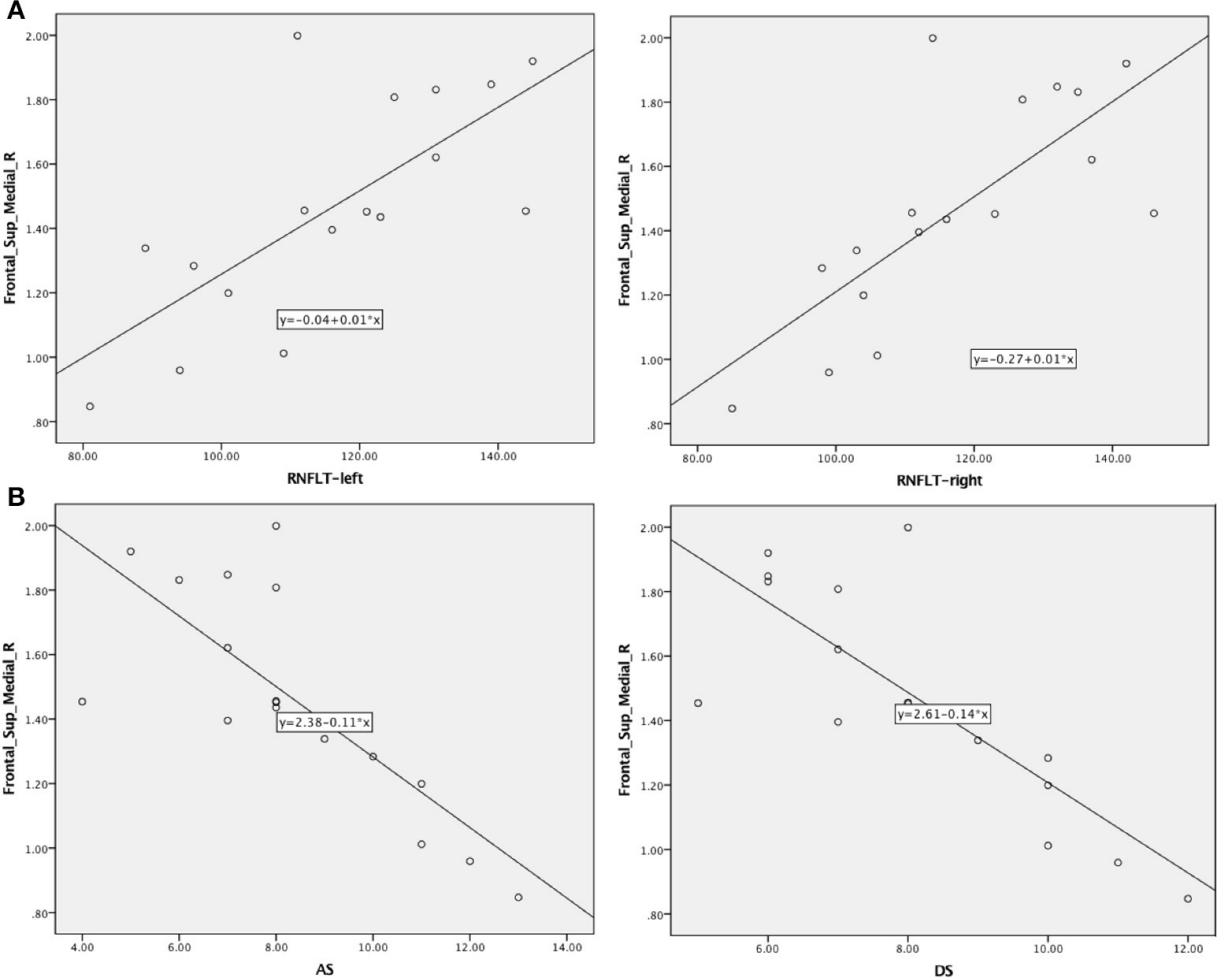
Correlations between AS/DS and RNFLT and the PerAF signal intensity in patients with MMD. **(A)** Negative associations were found between the AS/DS and the PerAF value in the Frontal_Sup_Medial_R. **(B)** Positive associations were found between left-RNFLT, right-RNFLT, and the PerAF value in Frontal_Sup_Medial_R. AS, anxiety score; DS, depression score; RNFLT, retinal nerve fiber layer thickness; PerAF, percent amplitude of fluctuation.

In patients with MMD, the RNFLT-left and RNFLT-right were positively associated with the PerAF value of the Frontal_Sup_Medial_R (*r* = 0.727, *p* < 0.01 and *r* = 0.748, *p* < 0.01, respectively) (Figure 4A, Table 3). In patients with MMD, the anxiety score (AS) and depression score (DS) were negatively associated with the PerAF value of the Frontal_Sup_Medial_R (*r* = −0.772, *p* < 0.01 and *r* = 0.806, *p* < 0.01, respectively) (Figure 4B).

**Table 3:** The results of the HADS test in patients with MMD and HCs.

**Patients**	**AS**	**DS**	**HCs**	**AS**	**DS**
Patient 1	7	7	HC 1	3	2
Patient 2	4	5	HC 2	3	2
Patient 3	6	6	HC 3	3	2
Patient 4	9	9	HC 4	3	1
Patient 5	8	8	HC 5	4	3
Patient 6	11	10	HC 6	4	2
Patient 7	10	10	HC 7	3	2
Patient 8	7	7	HC 8	1	2
Patient 9	12	11	HC 9	1	1
Patient 10	8	8	HC 10	1	2
Patient 11	8	8	HC 11	3	3
Patient 12	11	10	HC 12	3	2
Patient 13	7	6	HC 13	2	2
Patient 14	8	7	HC 14	3	2
Patient 15	13	12	HC 15	5	3
Patient 16	5	6	HC 16	3	1
Patient 17	8	9	HC 17	4	3

*MMD, moyamoya disease; HC, healthy control; HADS, Hospital Anxiety and Depression Scale*.

## Discussion

**Figure 5:**
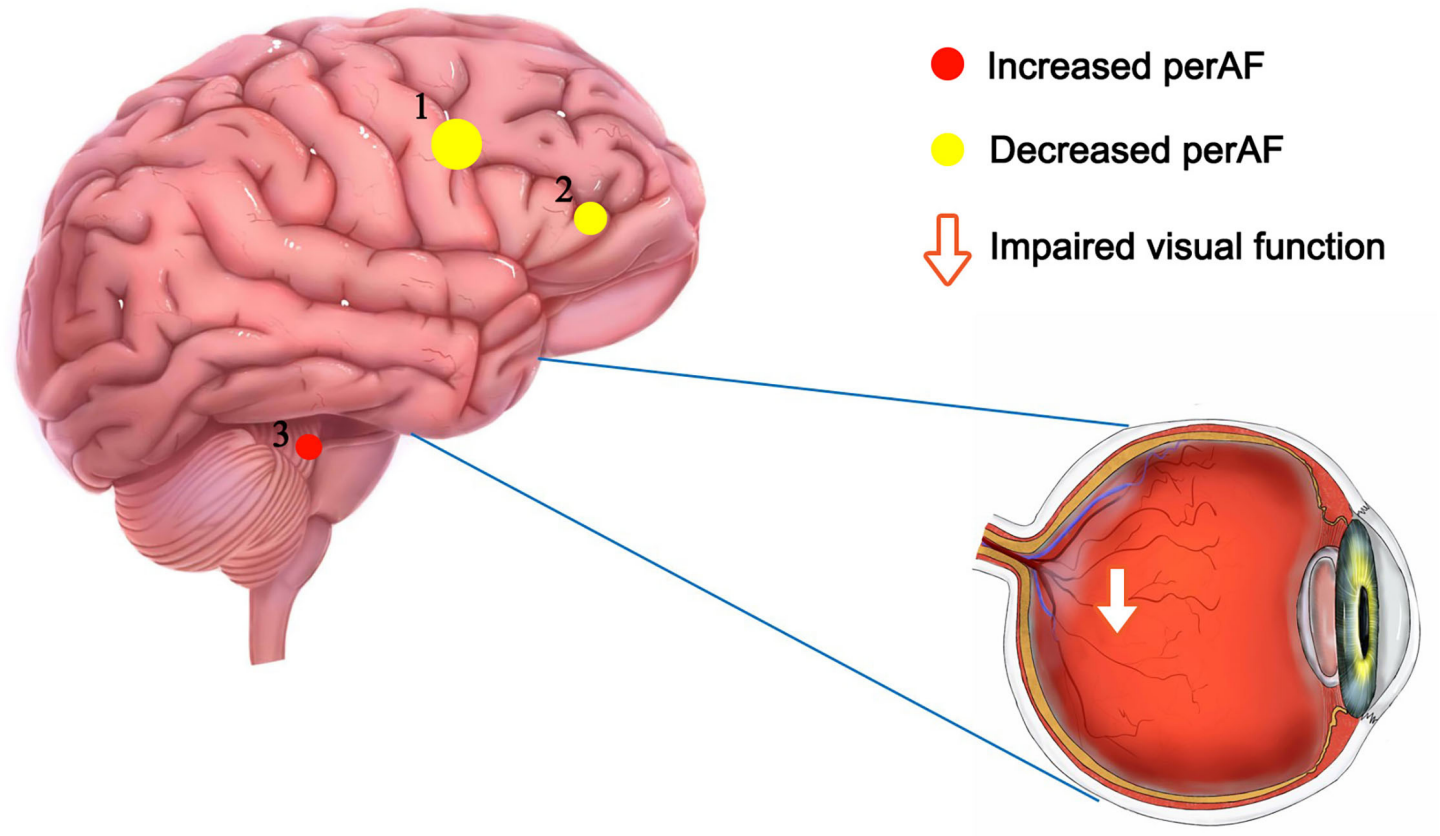
The mean perAF values of ROIs. Compared with the HCs, the PerAF values in Precentral_L (*t* = −4.3502) and Frontal_Sup_Medial_R (*t* = −3.8795) were increased and the PerAF value in Caudate_L (*t* = 3.8544) was decreased. PerAF, percent amplitude of fluctuation; HCs, healthy controls.

The PerAF reflects the resting-state BOLD signal fluctuations directly, it does not use arbitrary units, and is not influenced by raw signals, so the PerAF values are less affected by signal strength errors, more accurate, and more suitable for ensuing statistical analysis than other MRI analysis methods. The PerAF has been used in some studies of neurological disorders (Table 4) (14, 19–21), but this study is the first to use the PerAF to study changes in brain activity in a cerebrovascular disorder. The results showed that the PerAF values in MMD were decreased in Frontal_Sup_Medial_R and Precentral_L, but increased in Caudate_L compared with HCs (Figure 5).

**Table 4:** The PerAF methods applied in neurogenic diseases.

**PerAF method applied in neurogenic diseases**				
	**References**	**Disease**	**Brain areas**	
			**PerAF increased**	**PerAF decreased**
Neurogenic diseases	Wang et al. (20)	ET-1	B1-4: vermis VIII, B1-3: LCL VIII, B1-4: LPG	B5: vermis VIII, B4-5: LCL VIII, B5: LPG
	Zeng et al. (21)	Sleep Deprivation	BVC, BSC	BDPC, BCPL
	Yu et al. (19)	MCID	lLPHGs, TG	N/A

*ET-1, Type I epilepsy; B1-4, frequency band B1, B2, B3, B4; B1-3, frequency band B1, B2, B3; B5, frequency band 5; B4-5, frequency band B4, B5; LCL VIII, left cerebellar lobule VIII; LPG, left precentral gyrus; BVC, bilateral vision cortex; BSC, bilateral sensorimotor cortex; LPG, left para-hippocampus gyrus; MCID, mild cognitive impairment with depression symptom; TG, temporal gyrus; BDPC, bilateral dorsolateral prefrontal cortex; BCPL, bilateral cerebellum posterior lobe. PerAF, percent amplitude of fluctuation*.

The AS and DS from the HADS questionnaire were negatively associated with the PerAF in the Frontal_Sup_Medial_R in sufferers with MMD, indicating an association between MMD and psychiatric disorders. RNFLT value can be measured by OCT *in vivo* accurately and reproducibly, which provides high diagnostic ability in discriminating between healthy and glaucomatous eyes (22). RNFLT is now more and more commonly used in the staging system of glaucoma in the clinical practice. Previous studies demonstrated that the measurement of RNFLT provides important information for discriminating patients with ocular hypertension without any type of damage from those patients that do not show any functional damage, but have early structural glaucomatous signs (23, 24). The RNFLT-left and RNFLT-right were positively correlated with the PerAF value of the Frontal_Sup_Medial_R. Since we did not study the relationship between the PerAF and the course and progression of MMD, we are unable to determine whether RNFLT is related to the course of MMD. However, because changes in RNFLT are associated with various ocular diseases such as glaucoma, retinitis pigmentosa, ischemic optic neuropathy, optic neuritis, and cranial lesions that destroy or damage the optic nerve (25), the PerAF values as measured in this study can be used as an effective indicator of ocular complications associated with MMD. On the other hand, patients with MMD may have higher risk of developing eye diseases that are associated with abnormal RNFLT such as glaucoma, optic neuritis, and retinitis pigmentosa.

The superior frontal gyrus (SFG) is an important component of the third frontal lobe of the human brain and plays a crucial role in the regulation of movement, working memory, cognitive ability, self-awareness, and emotional regulation, in coordination with sensory systems (26). Fried et al. (27) found that electrical stimulation of the SFG triggered laughter in an epileptic patient. A higher level of electrical current increased the duration and intensity of laughter, from a smile at low currents to robust laughter with head waving at high current. The patient said her laughter was caused by the sensation of merriment and explained the laughter differently each time, attributing it to several external factors such as decor of the room or the content of the paragraph she was asked to read (27). The SFG is also involved in the perception and regulation of stress including acute psychosocial stress (28) and chronic life stress (29). Altered SFG gray matter volume was found in stress-related diseases such as major depression (30) and social anxiety disorder (31).

In this study, the PerAF was lower in the SFG of patients with MMD compared to controls. Combined with the above evidence, we deduce from our findings that sensory system impairment is likely in MMD, with reduced brain activity in the SFG. Therefore, patients with MMD may suffer from emotional disorders (depression and anxiety) resulting from decreased neural activity in the SFG. The risk of suffering from other disorders related to dysfunctions in the SFG such as cognitive disability and dyskinesia would also increase. The treatment of sufferers with MMD should include strategies for mood adjustment to avoid the development of depression and anxiety symptoms.

**Figure 6:**
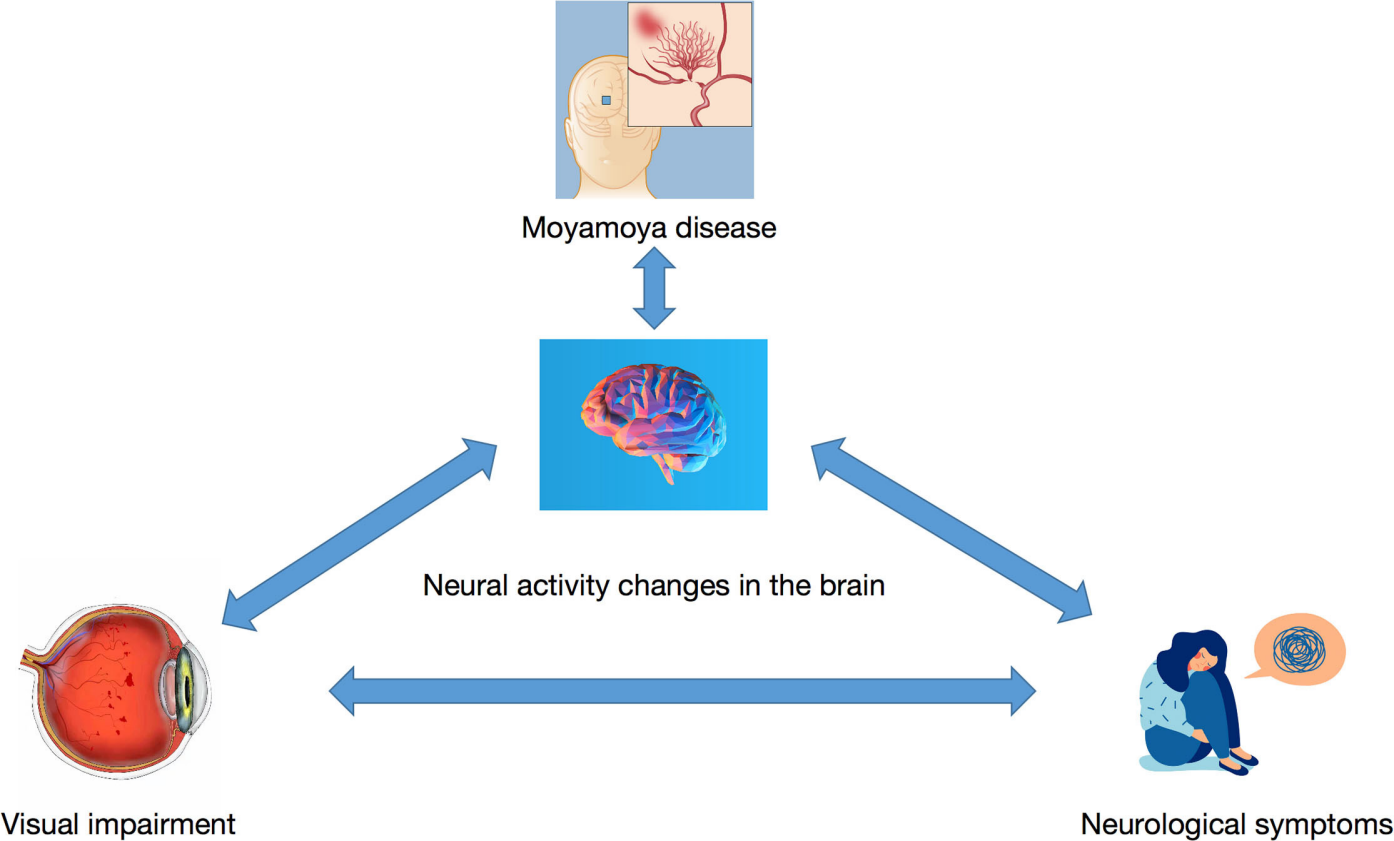
The relationship between MMD, mood changes, visual changes, and neural activity changes in related brain regions.

Furthermore, the mean PerAF value of the Frontal_Sup_Medial_R was negatively associated with the thickness of the left and right RNFL. Reduction in RNFLT is associated with various ocular diseases such as glaucoma, retinitis pigmentosa, ischemic optic neuropathy, optic neuritis, and cranial lesions that destroy or damage the optic nerve (25) (Figure 6). This may suggest that the PerAF analysis can be used as an early biomarker for ocular complications associated with MMD.

The precentral gyrus (PG) contains the primary motor cortex, which controls the regulation of voluntary movement of the contralateral side of the body (32). PG also comprises part of the supplementary motor cortex, which is responsible for the control of voluntary muscle contraction of the upper and lower limbs (33). When PG lesions occur, patients show dysfunction of the upper motor neurons with associated muscle weakness, abnormal muscle tone, contralateral paralysis (facial, leg, and arm paralysis), and pathological muscle stretch reflexes (such as Babinski sign) (34). Combining the above evidence with the decreased PG PerAF in this study, we can speculate that the extending terminal branches of the carotid artery may, to some extent, be compressed and cause PG dysfunction. The risks of contracting diseases related to PG, such as ataxia, contralateral paralysis, and dysmetria, would be increased in this situation.

The caudate nucleus is an important component of the corpus striatum, which controls spatial movement (35), spatial mnemonic processing, procedural learning (36), and associative learning (37). As part of the cortico-basal ganglia-thalamic loop, it also functions as a component of the reward system (38). The caudate nucleus receives input from the dopaminergic neurons projecting from the substantia nigra and from other associated brain regions. Previous studies indicate that the caudate nucleus is associated with the dysfunction of spatial working memory in patients with Alzheimer's disease as well as its abnormal dopamine supply to the striatum. Compared with non-spatial tasks, increased neural activity in the caudate nucleus is observed when undertaking tasks requiring spatial and motor memories (39). The caudate nucleus also takes part in regulating the speed and accuracy of directed body movements. Arushanian and Tolpyshev (40) removed the caudate nucleus in felines and observed defects in regulation of movement distance and accuracy. In another group in which the caudate nucleus was partially removed in felines, upper limb movement was delayed after stimulation, with constant adjustment of body posture and apparent failure to localize stimulation. A similar animal experiment conducted on monkeys involved the use of cocaine to damage the caudate nucleus. Uncontrolled forward movement and forward leaping were observed in the caudate-damaged monkeys, indicating that the caudate nucleus inhibits spontaneous forward movement when there is no resistance in the forward direction (41). The dorsal-prefrontal subcortical loop in the caudate nucleus is also associated with working memory. Hannan et al. (42) found in normal human subjects that the caudate nucleus is highly activated during working memory tasks, while in patients with schizophrenia, defects in working memory are linked to reduced innervation of the caudate nucleus. The amygdala sends projections to the caudate nucleus and they both have projections into the hippocampus. It is well established that the amygdala is important in memory processing. Lesions of connections between these structures obstruct the infusion of oxotremorine into the caudate nucleus, preventing its memory-enhancing effect and demonstrating the caudate nucleus function in memory processing in cooperation with amygdala. Combining the above evidence with the result of this study that higher PerAF signal was found in the caudate nucleus in sufferers with MMD, we may speculate that dysfunction in the MMD motor control system may cause compensatory overactivation of the caudate nucleus. Therefore, we hypothesize that increased neural activity in the caudate nucleus would cause disorders in motor control and memory learning, which is related to the function of this brain region. In clinical practice, interventions can be made to avoid these potential risks. Based on the above evidence, Table 5 illustrates the changed activation of brain regions in MMD, functions of those regions, and anticipated clinical outcomes.

**Table 5:** Brain regions alternation and its potential impact.

**Brain regions**	**Experimental result**	**Brain function**	**Anticipated results**
Frontal_Sup_Medial_R	MMD < HCs	Motor movement, working memory, cognitive ability, emotional regulation	Social and emotional problems, motor function defects, stress disorders and depression, cognitive disorders.
Precentral_L	MMD < HCs	Speech articulation, motor movement, planning of movement	Aphasia, muscle weakness, paralysis
Caudate_L	MMD > HCs	Motor processing, spatial mnemonic processing, procedural learning, associative learning, inhibitory control of action	Parkinson's disease, Alzheimer's disease, spatial movement disorder, defects in working memory, aesthetic obstacles, linguistic barrier

*MMD, moyamoya disease; HC, healthy control*.

In conclusion, changes of the PerAF signals are found in specific brain regions in sufferers with MMD, with decreases in the Frontal_Sup_Medial_R and Precentral_L and an increase in the Caudate_L. Patients with MMD may be at higher risk of diseases that are related to the dysfunction of these brain regions. This study had some limitations. For example, all the subjects were recruited from one province. As MMD is a relatively rare disease, the number of subjects that can be recruited is very limited and a relatively large and equal number of male and female subjects could not be included in this study. But since MMD is a rare disease, the incidence ranges from 1/280,000 to 1/89,000 in Japan and China to 1/1,100,000 in the US. The incidence of the disease in our country is much lower than that in the west. Thus, we have merely collected 46 cases with definite diagnosis and only 17 out of the 46 patients are willing to participate in this study. Therefore, the recruitment of experimental subjects has a certain degree of difficulty. Since our hospital is a well-known hospital in our country for treatment of MMD, patients come not only from the local, but from all over the country. Although it is a single-centered study, the subjects in this study are still representative. Therefore, the sample included large differences in the numbers of male and female participants. However, this study is the first study to use the PerAF to study neural activity changes in MMD. This study found altered brain activity in certain brain regions in sufferers with MMD and disclosed the neural mechanisms underlying clinical features such as anxiety and depression in patients with MMD. This study will also help to advance understanding of the pathogenesis of MMD and its complications, which will, in turn, lead to improvements in the diagnosis and treatment of MMD.

## Data Availability Statement

The raw data supporting the conclusions of this article will be made available by the authors, without undue reservation.

## Ethics Statement

The studies involving human participants were reviewed and approved by the Ethics Committee of The First Affiliated Hospital of Nanchang University. The patients/participants provided their written informed consent to participate in this study.

## Author Contributions

YS and E-MZ contributed to the guarantor of integrity of the entire study. H-YS and Y-CP contributed to the study concepts and design. Q-MG contributed to the literature search. TS and J-LW contributed to the clinical studies. L-JZ and C-QL contributed to the experimental studies/data analysis. R-BL and X-LL contributed to the statistical analysis. C-QL contributed to the editing and preparation of manuscript. All authors contributed to the article and approved the submitted version.

## Funding

This study was supported by the National Natural Science Foundation (No: 82160195), the Central Government Guides Local Science and Technology Development Foundation (No: 20211ZDG02003), the Key Research Foundation of Jiangxi Province (Nos: 20181BBG70004 and 20203BBG73059), the Excellent Talents Development Project of Jiangxi Province (No: 20192BCBL23020), the Natural Science Foundation of Jiangxi Province (No: 20181BAB205034), the Grassroots Health Appropriate Technology Spark Promotion Plan Project of Jiangxi Province (No: 20188003), the Health Development Planning Commission Science Foundation of Jiangxi Province (Nos: 20201032 and 202130210), and the Health Development Planning Commission Science TCM Foundation of Jiangxi Province (Nos: 2018A060 and 2020A0087).

## Conflict of Interest

The authors declare that the research was conducted in the absence of any commercial or financial relationships that could be construed as a potential conflict of interest.

## Publisher's Note

All claims expressed in this article are solely those of the authors and do not necessarily represent those of their affiliated organizations, or those of the publisher, the editors and the reviewers. Any product that may be evaluated in this article, or claim that may be made by its manufacturer, is not guaranteed or endorsed by the publisher.
